# The use of chilled condensers for the recovery of perfluorocarbon liquid in an experimental model of perfluorocarbon vapour loss during neonatal partial liquid ventilation

**DOI:** 10.1186/1475-925X-6-19

**Published:** 2007-05-31

**Authors:** Kimble R Dunster, Mark W Davies, John F Fraser

**Affiliations:** 1 Institute of Health and Biomedical Innovation, Queensland University of Technology, Brisbane, Queensland, Australia; 2 Grantley Stable Neonatal Unit, Royal Women's Hospital, Brisbane, Queensland, Australia; 3 Dept of Paediatrics & Child Health, The University of Queensland, Brisbane, Queensland, Australia; 4 Critical Care Research Group, Department of Intensive Care Medicine, The Prince Charles Hospital, Brisbane, Queensland, Australia

## Abstract

**Background:**

Perfluorocarbon (PFC) vapour in the expired gases during partial liquid ventilation should be prevented from entering the atmosphere and recovered for potential reuse.

This study aimed to determine how much PFC liquid could be recovered using a conventional humidified neonatal ventilator with chilled condensers in place of the usual expiratory ventilator circuit and whether PFC liquid could be recovered when using the chilled condensers at the ventilator exhaust outlet.

**Methods:**

Using a model lung, perfluorocarbon vapour loss during humidified partial liquid ventilation of a 3.5 kg infant was approximated. For each test 30 mL of FC-77 was infused into the model lung. Condensers were placed in the expiratory limb of the ventilator circuit and the amounts of PFC (FC-77) and water recovered were measured five times. This was repeated with the condensers placed at the ventilator exhaust outlet.

**Results:**

When the condensers were used as the expiratory limb, the mean (± SD) volume of FC77 recovered was 16.4 mL (± 0.18 mL). When the condensers were connected to the ventilator exhaust outlet the mean (± SD) volume of FC-77 recovered was 7.6 mL (± 1.14 mL). The volume of FC-77 recovered was significantly higher when the condenser was used as an expiratory limb.

**Conclusion:**

Using two series connected condensers in the ventilator expiratory line 55% of PFC liquid (FC-77) can be recovered during partial liquid ventilation without altering the function of the of the ventilator circuit. This volume of PFC recovered was just over twice that recovered with the condensers connected to the ventilator exhaust outlet.

## Background

Perfluorocarbon (PFC) liquids seem to be physiologically ideal for liquid ventilation, yet have two major drawbacks – economic and environmental. Being photochemically stable, PFC liquids have a high global warming potential [1] and contribute to the greenhouse effect [2]. It would seem prudent to limit PFC loss during partial liquid ventilation (PLV), both to prevent PFC vapour entering the atmosphere and to reuse the recovered PFC liquid. As all the lost PFC is in the exhaled gases, an opportunity exists to recover the PFC.

We have previously shown that up to 95% of PFC liquid can be recovered from vapour using chilled condensers [3,4] in place of an expiratory ventilator circuit. These studies, however, did not employ a ventilator and tidal breathing as would be found during clinical use of partial liquid ventilation. Also, the use of a model lung which is being ventilated by a neonatal ventilator presents the possibility of using the condensers attached to the ventilator exhaust outlet rather than in place of the expiratory circuit.

The aims of this study were: to determine how much PFC liquid could be recovered from expired gases (containing an approximate amount of perfluorocarbon vapour that would be found during partial liquid ventilation) using a conventional neonatal ventilator with chilled condensers in place of the usual expiratory ventilator circuit; to determine if PFC liquid could be recovered when using the chilled condensers at the ventilator exhaust outlet; and to compare the amount recovered with the two methods.

## Methods

**Figure 1:**
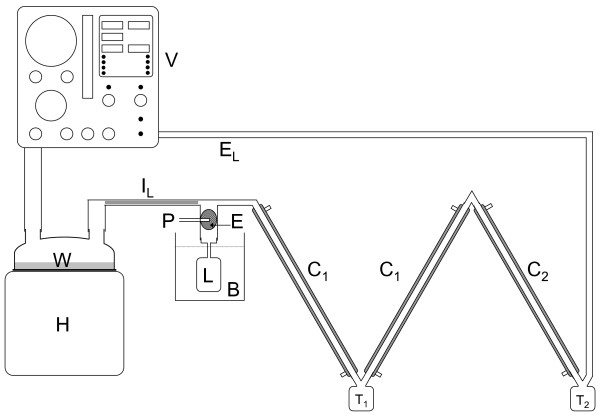
**Apparatus configured with condensers as the expiratory line**. V = ventilator, H = humidifier, W = water, P = perfluorocarbon inlet, E = perfluorocarbon evaporator, I_L _= heated inspiratory line, E_L _= short expiratory line, L = model lung, B = 39°C water bath, C_1 _= 1°C condenser, C_2 _= -30°C condenser, T_1_, T_2 _= traps

**Figure 2:**
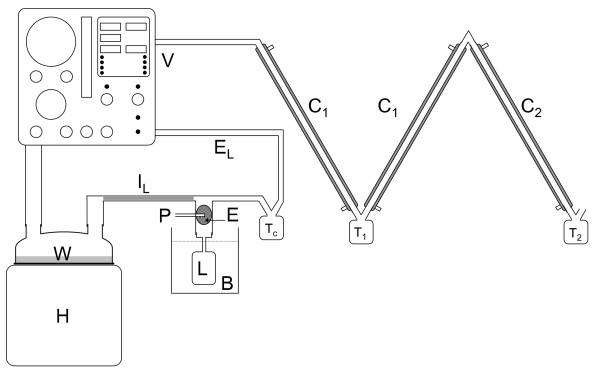
**Apparatus configured with condensers on the ventilator outlet**. V = ventilator, H = humidifier, W = water, P = perfluorocarbon inlet, E = perfluorocarbon evaporator, I_L _= heated inspiratory line, E_L _= short expiratory line, L = model lung, B = 39°C water bath, C_1 _= 1°C condenser, C_2 _= -30°C condenser, T_C_, T_1_, T_2 _= traps

We aimed to approximate the water and FC-77 concentrations found in the expired gases during partial liquid ventilation of an approximately 3.5 kg infant. A pressure-limited, time cycled neonatal ventilator (Bear Cub, Bear Medical Systems, Riverside, California) was set to deliver air at 60 breaths per minute with a positive end expiratory pressure of 5 cmH_2_O, a peak inspiratory pressure of 25 cmH_2_O, an inspiratory:expiratory ratio of 1:1 and a flow rate of 10 Lmin^-1^. A humidifier (MR600, Fisher & Paykel Healthcare, Auckland, New Zealand) was set to give a chamber outlet temperature of 39°C and an inspiratory limb outlet temperature of 37°C, as is commonly used in clinical practice. A self-filling chamber (MR290, Fisher & Paykel Healthcare, Auckland, New Zealand) and conventional heated inspiratory circuit (Fisher & Paykel Healthcare, Auckland, New Zealand) were used. A sponge (#00-005, Multigate Medical Products, Sydney, Australia) was placed in the outlet of the circuit manifold as an FC-77 evaporator. The circuit manifold, FC-77 evaporator and model lung were placed in a water bath (MD, Julabo, Seelbach, Germany) at 37°C. In this configuration, the lung had a tidal volume of 15.2 mL, an airway resistance of 140 cmH_2_OL^-1^s^-1 ^and a dynamic compliance of 0.9 mL/cmH_2_O. The set up of the experimental apparatus is shown in Figures 1 and 2.

For each test 30 mL of FC-77 (vapour pressure = 9.99 kPa at 37°C) [5,6] was infused onto the evaporator over a one hour period to approximate the loss rates found in animal studies [11]. At the conclusion of each test the condensers were warmed to ~2°C to ensure any water ice had melted, the volumes of water and FC-77 in each trap were measured and the total volumes calculated. The percentage of theoretical maximum recovery was calculated. The lung was inspected for the presence of FC-77. All measurements were performed five times.

### Use of condensers as the expiratory line

This configuration of the experimental apparatus is shown in Figure 1. The air, water vapour and FC-77 vapour from the lung passed through an expiratory limb consisting of two series-connected condensers and a short non-cooled section of tubing returning the gases to the ventilator. The first condenser consisted of two coaxial ventilator circuits (modified from prototype circuits, 10 mm internal diameter, smooth bore, 1 m long, Fisher & Paykel Healthcare, Auckland, New Zealand), with a total condensing surface of 0.063 m^2^, cooled to 1°C (2219 Thermostatic Circulator, LKB, Bromma, Sweden) [3,4] The second condenser consisted of a 1 m length of stainless steel tubing (13 mm internal diameter, 1 mm wall thickness) with a condensing surface of 0.041 m^2^, cooled to -30°C (9102 Circulator, Polyscience, Niles, Illinois, USA) [3,4].

### Use of condensers on the ventilator outlet

This configuration of the experimental apparatus is shown in Figure 2 and is the same as above, with the following changes. A conventional expiratory limb and trap (Fisher & Paykel Healthcare, Auckland, New Zealand) were used. The foam muffler was removed from the ventilator exhaust and the exhaust connected to the condensers. In addition to the circuit flow (10 Lmin^-1^) the ventilator discharges gas from a jet pump, used to overcome pressure drop in the expiratory limb, through the exhaust [7]. The total flow from the exhaust was measured at 16 Lmin^-1^.

### Theoretical maxima for condensation of water and perfluorocarbon

The gas stream would be saturated with water vapour which has a density of 44 mgL^-1 ^at 37°C and 5.2 mgL^-1 ^at 1°C [8]. Therefore a theoretical maximum of 38.8 mg of water could be condensed for each litre of this gas passing through the humidifier. This corresponds to a maximum hourly water recovery of 23.4 mL at 10 Lmin^-1 ^when the condensers are used as the expiratory limb.

When the gas stream that is saturated with water vapour is diluted with dry air at the ventilator exhaust the maximum hourly water recovery is reduced (to 21.6 mL) because the total flow from the exhaust is now 16 Lmin-1 and the amount of evaporated water remains constant in a larger volume of gas therefore less water is able to be condensed.

The saturated vapour density of FC-77 is 1.6 gL^-1 ^at 37°C, 0.28 gL^-1 ^at 1°C and 0.04 gL^-1 ^at -30°C [5]. In these experiments the vapour was not saturated and the pre-condenser vapour density was calculated by dividing the weight of FC-77 infused (53.4 g) by the total volume of air passed during the experiment. The condensers cannot reduce the vapour density below the saturation vapour density at the condenser temperature, dictating the maximum amount of FC-77 that can theoretically be condensed. The maximum possible hourly FC-77 recovery was 16.6 mL when the condensers are used as the expiratory limb and 8.52 mL when the flow was diluted with dry air at the ventilator exhaust.

### Statistical Analysis

Summary values are presented as mean (± SD). The difference between means is tested for statistical significance using Student's t-test (with Welch's correction where appropriate). A p value of < 0.05 was considered to represent a statistically significant difference.

## Results

No FC-77 was present in the model lung at the end of any experiment.

When the condensers were used as the expiratory limb of the ventilator circuit the mean (± SD) volume of water recovered was 20.8 mL (± 1.65 mL) and the mean (± SD) volume of FC-77 recovered was 16.4 mL (± 0.18 mL). The mean (± SD) proportion of infused FC-77 recovered was 54.8 (± 0.60) percent. These volumes recovered correspond to 89.1% (± 7.06%) of the theoretical maximum recovery for water and 99.0% (± 1.08%) for FC-77.

When the condensers were connected to the ventilator exhaust outlet the mean (± SD) volume of water recovered was 20.4 mL (± 0.96 mL) and the mean (± SD) volume of FC-77 recovered was 7.6 mL (± 1.14 mL). The mean (± SD) proportion of infused FC-77 recovered was 25.4 (± 3.80) percent. This corresponds to 94.6% (± 4.42%) of the theoretical maximum recovery for water and 89.3% (± 13.4%) for FC77.

The volumes of water (p = 0.65, t-test) and the percentage of maximum theoretical recovery (p = 0.17, t-test) did not vary depending on the condenser configuration. The volume of FC-77 recovered was significantly higher when the condenser was used as an expiratory limb (p < 0.0001, t-test with Welch's correction), but the percentage of maximum theoretical recovery did not differ with the condenser configuration (p = 0.18, t-test with Welch's correction).

## Discussion

For environmental and economic reasons it is beneficial to minimise the loss of PFCs during both partial and total liquid ventilation. PFCs do not support bacterial growth, biological materials will not dissolve in them and no chemical decomposition occurs at body temperature. As such, any recovered PFC can easily be washed and filtered for reuse in the same patient. All PFC loss occurs through evaporation and the PFC vapour all passes through the expiratory limb of the ventilator circuit and then to the ventilator exhaust. This provides two opportunities to condense the PFC vapour for reuse. Both perfluorooctyl bromide (vapour pressure = 1.47 kPa at 37°C) [6] and FC-77 (vapour pressure = 9.99 kPa at 37°C) [5,6] can be condensed during partial or total liquid ventilation.

The amount of vapour passing into the exhalation line during PLV depends on factors such as the surface area of the PFC-air interface, temperature and flow rate. Evaporative loss rates of 1.1 to 6 mlkg^-1^hr^-1 ^have been reported for partial liquid ventilation using perfluorooctyl bromide [9-12]. Higher loss rates, of around 7 mlkg^-1^hr^-1^, have been reported for the more volatile FC-77, which was used in these experiments [13]. Thus, over 10 L of PFC might be lost in an adult patient every day.

These same factors will effect condensation of the PFC vapour. The vapour pressure and, hence, the vapour density, of the PFC will impact on the efficiency of any condensation system. For example, with a lower vapour pressure than FC-77, less perfluorooctyl bromide is lost during ventilation and is more readily condensed (the condensability of PFC vapour increasing as vapour pressure decreases).

Using a neonatal ventilator with tidal breathing from a model lung, we studied the recovery of FC-77 with a loss rate of 30 mlhr^-1^, corresponding to the evaporative loss rate expected for an approximately 3.5 kg infant. Lower loss rates would be expected with the use of using perfluorooctyl bromide. Two configurations of the ventilator and condensers were studied. Firstly, the condensers were used as an expiratory limb of the ventilator circuit and secondly the exhaust from the ventilator was passed through the condensers to the atmosphere.

Two series-connected condensers were employed, the first at 1°C to remove water (which would solidify in the second condenser) and the second at -30°C to condense the FC-77.

Water is readily condensed at 1°C and over 89% of the theoretical maximum [8], was recovered in both condenser configurations. A significant amount of water was found in the second (-30°C) condenser – ~3 mL when the condenser were used as the expiratory limb and ~7 mL when the condensers were on the ventilator exhaust. This water forms ice in the second condenser and may block the condenser. In clinical practice it would be necessary to have two condensers which could be used alternatively to allow the ice to melt. Safety 'blow-off' valves and alarms would also be required to prevent excessive circuit pressures in the event of condenser blockage. An alternative strategy may be to use a scavenging system similar to that used to draw nitric oxide away from ventilator exhausts. The scavenged gases would then pass through the condenser. This strategy has the advantage of not connecting directly to the ventilator exhaust, but also has the disadvantage of further diluting the gases with room air.

In absolute terms, 55% of the evaporated PFC was recovered with the condensers as the expiratory limb and 25% with the condensers on the ventilator exhaust. When taken as a percentage of the theoretical maximum, 99% was recovered with the condensers as the expiratory limb and 89% with the condensers on the ventilator exhaust. A previous study using the same condensers showed recovery of 50% (or 86% of the theoretical maximum) of FC-77 at 10 Lmin^-1 ^using the same loss rate [4].

Schrader et al obtained a 90% recovery rate for perfluorooctyl bromide in animal studies utilizing PLV [14] by using a complicated rebreathing system. Specially designed heat and PFC exchangers fitted to the endotracheal tube may be also used to minimize PFC loss during PLV, with a conservation efficiency of 60% for perfluorooctyl bromide [15]. The high dead space in these devices would make them unsuitable for neonatal ventilation. Additionally, these methods require highly specialized ventilator circuits or other equipment. We have designed an expiratory circuit condenser with a small surface area, which does not significantly alter conventional ventilator function and achieves PFC recovery similar to other methods. The only additional equipment required are two circulating chillers.

Further cooling of the second condenser would increase the recovery. Cooling to dry ice temperature (-78°C) would increase the theoretical maximum recovery of FC-77 to >99% of the infused volume with the condensers as the expiratory limb and 98% with the condensers on the ventilator exhaust.

Ventilator settings suitable for neonates were studied and further studies will be necessary to determine the PFC recovery that can achieved with ventilators and circuits suitable for paediatric and adult ventilation.

## Conclusion

Using two series connected condensers in the ventilator expiratory line 55% of PFC liquid (FC-77) can be recovered from exhaled gas containing an approximate amount of perfluorocarbon vapour that would be found during partial liquid ventilation of newborn infants, without altering the function of the of the ventilator circuit. This volume of recovered PFC was just over twice that recovered with the condensers connected to the ventilator exhaust outlet where only 25% of PFC liquid (FC-77) could be recovered. In both cases the amount of PFC recovered was ≥ 89% of the theoretical maximum recovery.

## Competing interests

The author(s) declare that they have no competing interests.

## Authors' contributions

KRD conceived the study, participated in the design of the study, carried out the benchtop studies, performed the statistical analysis and drafted the manuscript. MWD conceived the study, participated in its design and coordination, assisted in the statistical analysis and revised the manuscript. JFF assisted in data analysis and interpretation, and revised the manuscript. All authors read and approved the final manuscript.
